# Hepatitis B vaccine coverage in health care students: a cross-sectional study in Vietnam

**DOI:** 10.1371/journal.pone.0320860

**Published:** 2025-03-31

**Authors:** Ly Trieu Vo, Dung Quoc Phan, Huy Gia Tran, Lam Thi Phuong Nguyen, Araba Gyan, Han Thi Ngoc Nguyen, Giao Huynh

**Affiliations:** 1 Faculty of Medicine, University of Medicine and Pharmacy at Ho Chi Minh City, Ho Chi Minh City, Vietnam; 2 Hospital for Tropical Diseases, Ho Chi Minh City, Vietnam; 3 Faculty of Control Disease, Health Center District 8, Ho Chi Minh, Vietnam; 4 Cardiology Department, Cho Ray Hospital, Ho Chi Minh City, Vietnam; 5 University of Michigan School of Public Health, Michigan, United States of America; 6 Infection Control Department, University Medical Center Ho Chi Minh City, Ho Chi Minh City, Vietnam; 7 Faculty of Public Health, University of Medicine and Pharmacy at Ho Chi Minh City, Ho Chi Minh City, Vietnam; National Research Centre, EGYPT

## Abstract

Hepatitis B virus (HBV) remains a significant global health concern, with healthcare students being at elevated risk of infection during their internships in healthcare settings. This study aimed to determine the percentage of healthcare students vaccinated against hepatitis B and the factors associated with vaccination status. A cross-sectional study was conducted among healthcare students at the University of Medicine and Pharmacy at Ho Chi Minh City in Vietnam between February and May 2023 using a self-reported questionnaire. Participants were selected through stratified and random sampling. A multivariable analysis logistic regression was performed to determine the association between several factors with vaccination status. A total of 225 participants took part in the study. We found that 89.8% of the participants had received at least one dose of the HBV vaccine, while 63.3% had completed the entire vaccine schedule. Students with sufficient knowledge of HBV were 2.68 times more likely to be vaccinated (p <  0.05), while those practicing effective HBV prevention had 8.79 times higher vaccination rates (p <  0.001) compared to others. The rate of HBV vaccination among healthcare students remains suboptimal. Targeted health education that addresses knowledge gaps, enhances motivation, tackles vaccination barriers, and emphasizes preventive measures before internships could substantially improve vaccination coverage in this group.

## Introduction

Hepatitis B (HBV) is a DNA virus that causes hepatitis in humans. It is transmitted through blood, sexual intercourse, and from mother to child. The transmission rate of HBV is 50-100 times higher than that of HIV [1]. The highest concentration of HBV in body fluids is found in blood, which is the most common way of transmission in healthcare settings [2]. Chronic HBV infection is a leading contributor to the incidence of cirrhosis, hepatocellular carcinoma, and ultimately fatal outcomes [3]. However, many individuals infected with HBV may not experience any symptoms [4]. As a result, there is a possibility that they may be unaware of their infection.

HBV is currently a major global health issue [4]. The World Health Organization (WHO) reports that the number of people living with HBV in 2022 was nearly 254 million, or 3.2% of the world population [5]. Global Health Systems Solutions is calling for the eradication of viral hepatitis as a public health concern by 2030 [4]. Vietnam is one of 10 countries contributing to almost two-thirds of the global burden of viral Hepatitis B, with the prevalence of HBV at 6.6% in 2022 [5,6]. By 2025, the number of people who will die from HBV is estimated to be around 58,600, which is a 60% increase from the figures recorded in 2005 in Vietnam [7].

Vaccination is the most important strategy for controlling HBV infection [2,8,9]. The Hepatitis B vaccine is 80% to 100% effective in preventing infection or progression to clinical hepatitis in people who receive the full dose [8]. There is evidence that routine vaccination and preventive measures reduced the number of HBV infections by 98% between 1983 and 2010 among healthcare workers (HCWs) in the United States [10].

Statistics indicate that sharp objects injure 5% to 35% of students participating in medical facility internships [11–13]. Among these injury cases, two-thirds are believed to be at risk of HBV exposure [14]. Hepatitis B has long been recognized as one of the occupational risks of HCWs because the infection risk is higher than that of the general population [2]. In 2021, a systematic review, including 89 articles, was conducted in developing countries and showed that only 52% of healthcare students receive at least 1 dose of Hepatitis B vaccination [15]. This proportion is lower than in Vietnam (83.9%) [16], Turkey (91.8%) [17], and Indonesia (59%) [18] and some developed countries like India (72.5%) [19], and China (86%) [20]. In Nepal, the majority of healthcare students are familiar with the fact that the HBV vaccination schedule consists of at least three doses (82.5%) and the full course of the vaccine is approximately 95% effective (86.9%) [21].

Several factors are relevant to students’ HBV vaccination, including their knowledge, attitude, and practice to prevent HBV, as well as their major, sex, academic year, department, and childhood residence [12,17,22–25]. Other factors such as student income, availability, and pricing of the Hepatitis B vaccine vary between countries, which can lead to differences in HBV vaccination rates among students [15,17,23]. In Turkey, 4.4% of students have not received the Hepatitis B vaccine due to its elevated cost [17], whereas in Ghana, this figure reaches 30.3% [23]. Limited research exists on the barriers and assesses the impact of economic status on student HBV vaccination in Vietnam.

In Vietnam, there are no regulations requiring healthcare students to receive the HBV vaccine before admission and internship in healthcare settings, neither is the vaccine provided for free. Therefore, Hepatitis B vaccination is entirely based on the student’s intention to receive it. Furthermore, the lack of knowledge and experience in medical practice during internships can lead to an increased risk of sharps injuries [26,27] thereby increasing their susceptibility to Hepatitis B infection [14,27]. In this study, we aim to determine the percentage of students vaccinated against HBV and the factors associated with vaccination status. Additionally, we propose appropriate measures to improve the vaccine coverage rate.

## Materials and methods

### Study design and setting

A cross-sectional study was conducted at the University of Medicine and Pharmacy at Ho Chi Minh City, Vietnam (UMP). The study was conducted across 5 faculties, namely the Faculty of Medicine (Med), Nursing and Medical Technology (NMT), Public Health (PH), Odonto-Stomatology (OS), and Traditional Medicine (TM). The participants for this research were final-year healthcare students, in other words, 6th-year students for 6-year majors and 4th-year students for 4-year majors. The inclusion criteria were final-year students who were studying at UMP from February to May 2023 and consented to the study. There were 1,962 students, out of those 345, 147, 872, 194, and 404 were in PH (17.8%), OS (7.5%), NMT (44.4%), TM (10.0%), and Med (20.1%), respectively. The exclusion criteria were healthcare students who were absent more than 2 times for the study.

### Sample size and sampling procedure

A total of 1,962 final-year healthcare students. A single population proportion formula was used with a 95% confidence interval ($Z_{\alpha /2}$ = 1.96), a 5% margin of error, and population proportion (p) was referenced from research conducted in Vietnam [16]. We calculated a minimum sample size of 208 students, accounting for a 10% sample loss, with a final sample size as 229. We utilized the stratified sampling method to allocate the number of students from each faculty. The total sample size of 229 was distributed proportionally to each department based on their student population. Sample sizes of 40, 18, 97, 28, and 46 were allocated to PH (17.5%), OS (7.8%), NMT (42.3%), TM (12.1%), and Med (20.1%), respectively. Researchers contacted the faculty training management to request a list of students to select research participants. The students were sorted into their respective faculties and numbered in ascending order according to the list. The researcher then used the random.org website to randomly select participants based on the number of students from each department.

### Instrument

Data were collected using a self-reported questionnaire referenced and built on previous studies [16,17,23], and the domains of the Health Belief Model [28]. The questionnaire gathered information on sociodemographic characteristics, knowledge, and practices related to preventing HBV, and history of Hepatitis B vaccination. The structured questionnaire was divided into four parts, comprising a total of 25 close questions. The first part, consisted of 7 questions, collecting sociodemographic characteristics such as sex, faculty, economic dependence on family, how students perceive their family’s economic status, whether family members are healthcare workers, history of contact with patients diagnosed with Hepatitis B (including medical examinations, care, and medical procedures) and history of injuries caused by sharp objects during the internship. The second part had 3 questions aimed at determining the history of HBV vaccination, motivation, and barriers to receiving the Hepatitis B vaccine.

The third section included 10 questions to evaluate students’ knowledge about HBV prevention, covering six aspects: the dangers of HBV, transmission, symptoms, treatment, prevention, and the HBV vaccine. The fourth section consisted of 5 questions assessing HBV-related practices, including timing of vaccination, HBV screening tests, wearing gloves, recapping needles after use, and safe disposal of sharps [16,17,23].

Multiple-choice questions were administered to evaluate healthcare students’ knowledge and practices related to HBV prevention. For both the knowledge and practice sections, participants received one point for each correct response, while incorrect or “I don’t know” answers received zero points. The questionnaire was initially administered in a pilot study involving 23 volunteer students from five faculties, recruited through social media. The questions were tested for clarity, understandability, and appropriateness, with modifications made after the pilot study. Participants in the pilot study were excluded from the main study.

### Operational definition

#### HBV vaccination history.

Participants were categorized into two groups: those who had received full doses of HBV vaccine ( ≥ 3 doses) and those who had not received full doses of HBV vaccine (less than 3 doses).

#### Knowledge.

Participants were divided into two groups based on their total score: Those with a score of 70% or higher were considered to have “sufficient knowledge”, while those with a score lower than 70% were categorized as having “insufficient knowledge” [12,16,29].

#### Practice.

Participants were divided into two groups based on their total score: Those with a score of 70% or higher were considered to have “good practice”, while those with a score lower than 70% were categorized as having “not good practice” [12,16,29].

### Data collection

The participants were recruited with the help of class leaders. Students who were absent for the first time were contacted again at a later time. There were 2 investigators assisting with data collection. All investigators were healthcare workers who had been trained before data collection. Students were presented with consent forms along with how to answer questions ensuring accuracy and comprehensiveness of the data. Participants had the right to ask the investigators questions to ensure that the questions were understood as intended. It took 15 - 20 minutes to complete the questionnaire. After participants completed the survey, the questionnaire was collected and checked by the investigators.

### Data analysis

Data was entered by using Epidata 3.1 and analyzed using STATA 17. All qualitative variables were expressed as numbers (n) and percentages (%). The dependent variable is the student’s history of HBV vaccination. Independent variables include sociodemographic characteristics (sex, faculty, economic dependence on family, perceived family economic status, whether family members are healthcare workers, history of contact with a hepatitis B patient, and history of injuries caused by sharp objects), knowledge, and practice to prevent Hepatitis B. We used both univariate and multivariate analyses to demonstrate the association between full vaccination status and independent factors. In the univariate analysis, we used the Chi-square test to select the eligible variables for the multivariate analysis. Any variable with a p-value of less than 0.2 was considered eligible for the multivariate analysis [30]. The multivariate analysis used the multivariate logistic regression method, with odds ratio (OR) and 95% confidence interval (95% CI). Significance was considered as a p-value less than 0.05.

### Ethical issues

The research was approved by the Ethics Committee in Biomedical Research of the University of Medicine and Pharmacy at Ho Chi Minh City (No 73/HDDD on 16 January 2023). Before beginning the study, the researcher explained the purpose of the research to the students, how their data will be used, and address any questions they may have. Students who agreed to participate were asked to sign a consent form to confirm their voluntary participation. Following this, the researcher conducted the survey using a structured questionnaire. As part of the informed consent process, participants were informed that they were not required to answer any questions they found objectionable and had the right to withdraw at any time. The questionnaire did not collect any identifying information about the participants.

## Results

### Characteristics of study participant

The study consisted of 225 healthcare students from five faculties, with a response rate of 96% (4 students were absent more than 2 times). The distribution of students from each faculty in the research was as follows: 42.7% from NMT (96), 20.0% from Med (45), 17.3% from PH (39), 12.4% from TM (28), and 7.6% from OS (17). 59.6% were women, and 40.4% were men. 79.6% of students indicated depended on their family’s finances. 81.6% perceived their family’s economic situation as sufficient to live on. About 76.6% of students reported that their family does not include a member who was HCWs. 80% had been in contact with hepatitis B patients before and 42.7% of the healthcare students suffered injuries caused by sharp objects during the internship. Of these, 67.7% experienced more than one injury (Table 1).

**Table 1:** The association between characteristics of healthcare students’ HBV vaccination (n = 225).

Character	Total n(%)	Full doses vaccination		p^*^
		Yes n = 142 (%)	No n = 83 (%)	
Sex				
Male	91 (40.4)	57 (62.6)	34 (37.4)	0.9
Female	134 (59.6)	85 (63.4)	49 (36.6)	
Faculty				
Medicine	45 (20.0)	34 (75.6)	11 (24.4)	0.14
Public health	39 (17.3)	23 (59.0)	16 (41.0)	
Traditional medicine	28 (12.4)	13 (46.4)	15 (53.6)	
Odonto-Stomatology	17 (7.6)	10 (58.8)	7 (41.2)	
Nursing and Medical Technology	96 (42.7)	62 (64.6)	34 (35.4)	
Depends on the family’s economic^&^	179 (79.6)	116 (64.8)	63 (35.2)	0.3
Perceived family’s economic (n = 223)				
Well off	22 (9.9)	14 (63.6)	8 (36.4)	0.6
Enough to live	182 (81.6)	113 (62.1)	69 (37.9)	
Not enough to live	19 (8.5)	14 (73.7)	5 (26.3)	
Family members were HCW^&^	170 (76.6)	110 (64.7)	60 (35.3)	0.3
Contact with a hepatitis B patient before^&^	180 (80.0)	116 (64.6)	64 (35.4)	0.4
Injured by sharp objects during intern				
Yes	96 (42.7)	62 (64.6)	34 (35.4)	0.7
More than one time (n = 96)	65 (67.7)	–	–	
One time (n = 96)	31 (32.3)	–	–	
No	129 (57.3)	80 (62.0)	49 (38.0)	
Sufficient knowledge	187 (83.1)	127 (67.9)	60 (32.1)	0.001
Good practice	173 (76.9)	129 (74.6)	44 (25.4)	<0.001

*The p-value was calculated via the Chi-square test,

& The Yes group.

In univariate analysis, 3 variables that included students from each faculty, knowledge, and practice to prevent HBV (p < 0.2) were entered into the multivariate analysis. There was no statistical significance between sex, personal and family economic status, career of family members, and history of exposure to HBV among students’ HBV vaccination (Table 1).

### HBV vaccination status of study participants

Regarding vaccination status, 202 students (89.8%) had received at least 1 dose of the HBV vaccine and 63.1% of participants had completed the vaccine series ( ≥ 3 doses). 83.6% were motivated to get vaccinated because they were aware of the occupational risk and the high probability of being infected with hepatitis B, and 53% thought that Hepatitis B is a dangerous disease. 10.2% of students had not been vaccinated at all. The main reasons were that vaccination is not mandatory (43.5%), they do not know where to get vaccinated (34.8%), and they do not have time (34.8%) (Table 2).

**Table 2:** HBV vaccination history of study participants at the University of Medicine and Pharmacy at Ho Chi Minh City, Vietnam (n = 225).

Vaccination	Number (n)	Percentage (%)
Number of HBV vaccine doses		
None	23	10.2
1 dose	16	7.1
2 doses	44	19.6
≥ 3doses	142	63.1
Full-dose vaccination against HBV		
Yes	142	63.1
No	83	36.9
Vaccination motivation (n = 202)		
High risk of hepatitis B	169	83.6
Because hepatitis B is dangerous	107	53.0
My teacher introduced	72	35.6
Family introduced	46	22.7
My friends recommend	34	16.8
Good role model for the family	33	16.3
Vaccination barriers (n = 23)		
Injections are not compulsory	10	43.5
Don’t know where to get the vaccine	8	34.8
Do not have time	8	34.8
Hard to get a hepatitis B infection	5	21.8
Concerned about vaccination costs	3	13.0
Vaccine side effects	2	8.7
Do not trust the hepatitis B vaccine	1	4.4

### Study Participants’ Knowledge of Hepatitis B Virus

Table 3 illustrates that 83.1% had sufficient knowledge about hepatitis B. 81% agreed that HBV can cause chronic infection, while 98% agreed that it can lead to cirrhosis, liver cancer, and finally could lead to death (95.1%). There were 79.1% of students who were fully aware of the symptoms of HBV infection. Regarding treatment, 88% of participants knew that chronic hepatitis B treatment was long-term. 96.4% of the students agreed that all adults, children, and infants should be vaccinated with the hepatitis B vaccine. 63.1% of students are correctly aware of the transmission routes of hepatitis B, and 69.3% agree that the majority of people have asymptomatic acute and chronic HBV infection. 32.4% of students do not have accurate knowledge about HBV prevention. 69.3% of participants knew about the high-risk groups who should be given priority for HBV (Table 3).

**Table 3:** Healthcare student’s knowledge of Hepatitis B at UMP, Vietnam (n = 225).

Knowledge	Sufficient	
**Number** **(n)**	**Percentage (%)**
HBV causes chronic infection	182	81.0
HBV causes cirrhosis and liver cancer	221	98.0
HBV causes illness and leads to death	214	95.1
HBV transmission	142	63.1
HBV infection can be asymptomatic	156	69.3
Symptoms of acute HBV infection	178	79.1
Chronic HBV treatment possibly for life	198	88.0
Behaviors prevent HBV	152	67.6
All adults, children, and infants should be vaccinated against HBV	217	96.4
The high-risk groups who should be given priority vaccination	156	69.3
**Knowledge** ^#^	**187**	**83.1**

# *The participants had a total score that indicates sufficient knowledge in the knowledge section.*

### Preventive practices against hepatitis B virus among study participants

According to our data, 76.9% of students had good practices preventing HBV infection. Up to 91.6% of students regularly wear gloves, and 84.9% regularly dispose of sharp objects in the appropriate container after use. However, only 76% of students screened for HBV, and 76.4% vaccinated against HBV on their own before participating in practice at the clinical centers. About 41.8% frequently cover the syringe with 2 hands after use (Table 4).

**Table 4:** Healthcare student practice prevents Hepatitis B at UMP, Vietnam (n = 225).

Practice	Good	
**Number (n)**	**Percentage (%)**
HBV Vaccinations before internship	172	76.4
Hepatitis B screening test before Internship	171	76.0
Always wear gloves when coming into contact with blood, secretions, or patients	206	91.6
Regularly put sharp objects in the container immediately after use	191	84.9
Do not use two hands to cover the syringe with a needle cap	131	58.2
**Practice** ^#^	**173**	**76.9**

# *The participants had a total score that indicates good practice in the practice section.*

### Factors associated with HBV vaccination among study participants

In our multivariable analysis, the full dose HBV vaccination was 2.68 times (OR = 2.68, 95% CI 1.13 – 6.35, p = 0.025) higher in students with sufficient knowledge than those with insufficient knowledge. The vaccination was 8.79 times higher (OR = 8.79, 95% CI 3.92 – 19.73, p <  0.001) in participants with good preventive practice than in those without good practice. The faculty had no association with the HBV vaccination of students **(**Table 5***).***

**Table 5:** The result of multivariable analysis between factors related to the full HBV vaccination (n = 225).

Variables	Full doses vaccination		OR (95% CI)	p^**^
	Yes (n = 142)(%)	No (n = 83)(%)		
Faculty				
Medicine	34 (75.6)	11 (24.4)	1	0.69
Public health	23 (59.0)	16 (41.0)	0.86 (0.27 – 2.32)	0.49
Traditional medicine	13 (46.4)	15 (53.6)	0.66 (0.20 – 2.15)	0.15
Odonto-Stomatology	10 (58.8)	7 (41.2)	0.39 (0.10 – 1.43)	0.41
Nursing and Medical Technology	62 (64.6)	34 (35.4)	0.68 (0.26 – 1.72)	0.69
Knowledge				
Sufficient	127 (67.9)	60 (32.1)	2.68 (1.13 – 6.35)	**0.025**
Insufficient	15 (39.5)	23 (60.5)	1	
Practice				
Good	129 (74.6)	44 (25.4)	8.79 (3.92 – 19.73)	**<0.001**
Not good	13 (25.0)	39 (75.0)	1	

*Faculty, student knowledge, and practice were included in a multivariate analysis;*

**the p-value was calculated using multivariate logistic regression.

## Discussion

This cross-sectional study conducted in Vietnam in 2023 aimed to determine the HBV vaccination rate among healthcare students and its associated factors. Only two-thirds of the participants had received the full dose of the vaccine. Students with sufficient knowledge and good preventive practices for HBV had a higher vaccination rate than those without.

### Coverage of HBV vaccine

The current study reveals that 89.8% of participants reported receiving at least one dose of the Hepatitis B Virus (HBV) vaccine, a percentage that aligns with similar studies conducted in Vietnam (83.9%) [16] and Turkey (91.8%) [17]. However, this vaccination rate is significantly higher than those reported in Ethiopia (25.7%) [12], Nepal (60.8%) [31], Ghana (44.4%) [23], and Uganda (66.8%) [32]. These discrepancies can be attributed to several factors, including differences in participant characteristics such as sex [12,17], years of education [12,32], and academic major [12,17,32]. The participants in this study were primarily final-year healthcare students, which may have contributed to the high vaccination rate observed.

Another factor contributing to the differences in vaccination coverage across countries is the variation in healthcare systems, particularly in terms of the cost and availability of the HBV vaccine [23,32]. These challenges highlight the importance of considering local healthcare contexts when designing and implementing vaccination campaigns. To effectively increase HBV vaccination rates among healthcare students, interventions must consider the diverse factors that influence vaccination behavior. This may include sex, academic major, years of education, and the economic barriers related to vaccine cost and availability. Tailoring vaccination campaigns to address these specific factors can lead to more equitable and widespread vaccine coverage, ultimately reducing the risk of HBV transmission in healthcare settings.

The insufficient focus on the reasons for healthcare students’ failure to receive the HBV vaccination, particularly in the context of Vietnam, is a significant concern. The present study has identified several barriers to vaccination among these students, underscoring areas that require intervention to improve HBV vaccine coverage. The primary reasons cited include the absence of a mandatory vaccination requirement (43.5%), insufficient information on where to obtain the vaccine (34.8%), and time constraints (34.8%). Additionally, 21.8% of students believed vaccination was unnecessary because they had not contracted HBV. These findings align with a systematic review of 89 articles conducted in developing countries, which identified similar barriers to vaccination [15]. A smaller percentage expressed concerns about vaccine safety, with 8.7% worried about potential side effects and 4.4% expressing distrust in the vaccine.

Interestingly, the cost of HBV vaccination was not a significant concern for most students in Vietnam, with only 13% indicating it as a reason for non-vaccination. Furthermore, there was no observed correlation between a student’s income or family economic status and their likelihood of receiving the HBV vaccine. This is in stark contrast to findings from studies in Ghana [23] and Uganda [32], where the high cost of the vaccine was a major barrier to HBV vaccination. The difference in these results may be attributed to the more affordable price of the HBV vaccine in Vietnam, priced at less than 13 dollars per dose, making it accessible to a broader range of students. The identified barriers to vaccination among healthcare students highlight several areas that need to be addressed to improve HBV vaccine coverage.

In Vietnam, healthcare students are not required to receive the HBV vaccine before admission or internships, nor is the vaccine provided for free. This group receives less attention, although studies indicate low HBV vaccine coverage among healthcare students. Vietnam’s Expanded Program on Immunization (EPI), launched in 1981, mandates free HBV vaccination for children under 5, with 98% receiving at least three doses by age 2, there is no similar mandate for healthcare students [33]. The other mandated vaccines in Vietnam EPI are whooping cough, diphtheria, tetanus, Haemophilus influenzae type b, polio, measles, rubella, Japanese encephalitis, and tuberculosis. We believe that requiring HBV vaccination for healthcare students before internships and ensuring accessible vaccination information could significantly improve coverage. Additionally, educational campaigns that highlight HBV vaccine safety and address common misconceptions could build trust and alleviate concerns. Since cost is not a primary barrier, efforts should focus on regulatory policies and improving awareness. Furthermore, health education should strongly emphasize recommendations from trusted healthcare providers and government authorities to encourage students to get vaccinated, particularly those undergoing training at hospitals. Specifically, the content should highlight the risks of HBV infection, the severity of the disease, and students’ susceptibility to potential illness. By raising awareness of these factors, vaccination acceptance among students can be significantly improved. Moreover, to enhance monitoring and ensure compliance, a well-structured software system should be implemented to systematically record vaccination histories and maintain corresponding databases. This system would not only help track students’ vaccination status but also facilitate targeted health education for those who have not yet been vaccinated. Additionally, universities should take proactive steps to offer convenient access to vaccination points, such as university clinics, in order to minimize logistical barriers and boost students’ confidence in getting vaccinated.

Approximately two-thirds of individuals who received the HBV vaccine reported receiving complete dosages (63.1%). This rate is higher than a similar report in Vietnam (30.3%) [22], Uganda (44.3%) [32], Ethiopia (5.8%) [12], and Ghana (30.5%) [23]. Sex, type of student (top-up students who combine prior certifications with additional years of full-time study, and regular students), major, academic year, perceived family economic, knowledge, and practice of student were demonstrated to be associated with a complete HBV vaccination series [12,17,23,32]. The variation of these factors between research may contribute to the difference in vaccine coverage. Finding a solution for students to supplement with HBV vaccination is necessary. Existing research indicates that common reasons for not completing the vaccination schedule include being too busy or forgetting to attend follow-up appointments [12]. Another reason could be the Lockdown due to COVID-19 in Ho Chi Minh City, Vietnam in 2021-2022 disrupted student vaccinations. One practical solution to increase adherence to the HBV vaccination schedule is for health centers to actively remind students of their follow-up doses through text messages or phone calls. Additionally, arranging more flexible vaccination schedules that accommodate students’ busy academic lives could further improve completion rates.

The current research revealed that 76.9% of participants adhered to recommended preventive measures. This rate is notably higher compared to findings from previous studies in Ethiopia [12], and Nepal [21], which reported adherence rates of 47%, and 33%, respectively. These disparities may be attributed to differences in the demographic and educational backgrounds of participants, as well as variations in the healthcare systems and training environments across these countries. The 2024 study from Nepal highlighted that the year of education, history of HBV testing, vaccination status, and direct encounters with HBV patients were significant predictors of adherence to Hepatitis B preventive measures [21]. Given these findings, healthcare students must receive comprehensive education on HBV prevention before commencing their internships.

### Association with HBV vaccination

This study found that 83.1% of participants had sufficient knowledge about Hepatitis B, a figure that is consistent with findings from similar studies conducted in Vietnam (89.2%) [16] and Turkey (83%) [17]. The analysis indicated that students with adequate knowledge were 2.68 times more likely to receive the full HBV vaccination dose compared to those with insufficient knowledge. This result is consistent with findings from studies conducted in Vietnam [16,24], China [20], and Turkey [17] which also identified a positive correlation between knowledge and Hepatitis B vaccination rates, which also demonstrated a positive correlation between knowledge and Hepatitis B vaccination rates. The current research question is based on the Health Belief Model proposed by Glanz Karen, et al., which highlights four key factors that influence individual behavior: perceived threats, benefits, barriers, and self-efficacy [28]. Healthcare students with sufficient knowledge of HBV are more aware of the disease burden, occupational risks associated with HBV, and the protective benefits of the HBV vaccine in a healthcare environment, which explains their higher vaccine coverage compared to their less-informed counterparts. Moreover, research on the general adult population in Vietnam found that even when participants were provided with free three-dose HBV vaccine vouchers, vaccination rates remained low [24]. This suggests that improving knowledge could enhance vaccine uptake. Consequently, a health education initiative grounded in the Health Belief Model, which targets gaps in HBV knowledge, could potentially increase vaccination rates among healthcare students [28].

Additionally, we found that students who practiced Hepatitis B prevention had a higher vaccination rate than those who did not have good prevention practices. This result is similar to the report in Nepal [21], China [20], Turkey [17], and Ethiopia [12]. The higher vaccination rates among these students may be attributed to their heightened awareness of the substantial risk posed by HBV infection. According to the WHO, HBV vaccination is recognized as the most effective measure for protecting healthcare workers in clinical settings [34]. To enhance HBV vaccination coverage among students, it is crucial to implement comprehensive pre-internship training programs that emphasize HBV prevention. Such programs should include not only vaccination but also testing for HBV, strategies to prevent sharp injuries, and the consistent use of protective gloves during clinical internships. These practices collectively contribute to reducing the risk of HBV infection and improving vaccination rates among healthcare students.

One of the strengths of this study is the inclusion of healthcare students from various majors, which provides a more comprehensive understanding of the factors influencing Hepatitis B vaccination among this diverse group. The study also offers valuable insights into the motivations, barriers, and associated factors related to HBV vaccination in healthcare students.

However, this study has some limitations. First, age information was not collected. Additionally, data on practices and participants’ HBV vaccination status were gathered through self-reporting, without verification via official documentation, due to the lack of a systematic software system for recording adult vaccination histories in Vietnam. This limitation makes it challenging to accurately verify HBV vaccination status. Consequently, vaccination records relied on self-reported data, as noted in previous studies, which may have introduced some bias. Furthermore, the sample used in this study may not fully represent the broader population of healthcare students, potentially limiting the generalizability of the findings.

## Conclusions

The persistently low rate of complete Hepatitis B vaccination among healthcare students presents a significant concern, given the critical importance of vaccination in preventing HBV infections, particularly in clinical settings where exposure risks are high. This study proposes that targeted health education that addresses gaps in knowledge, enhances motivation, tackles barriers to vaccination, and provides pre-practice HBV prevention internship, has the potential to significantly improve HBV vaccination coverage among healthcare students. We believe that such an intervention will not only increase vaccination rates but also foster a more informed and proactive approach to health protection among future healthcare workers. By equipping students with the necessary knowledge and practical experience, this research aims to ensure better protection for healthcare students and contribute to safer healthcare environments.

## Supporting information

S1 File HBV dataset.
(XLSX)
